# High Concentration of FBS Can Save mTOR Down-Regulation Caused by *Mycoplasmas bovis* Infection

**DOI:** 10.3390/vetsci9110630

**Published:** 2022-11-11

**Authors:** Xiaochun Wu, Jinrui Ma, Shangdong Jia, Xudong Zhang, Xinlan Zhang, Zhen An, Yanquan Wei, Xiaoyong Xing, Fengqin Wen, Yuan Gao, Shijun Bao

**Affiliations:** 1 College of Veterinary Medicine, Gansu Agricultural University, Lanzhou 730070, China; 2 College of Life Science and Technology, Gansu Agricultural University, Lanzhou 730070, China

**Keywords:** *Mycoplasma bovis*, mTOR signaling pathway, EBL cells, nutritional metabolism, bovine serum

## Abstract

**Simple Summary:**

*Mycoplasmas bovis* is a devastating pathogen in dairy cows worldwide and is responsible for substantial economic health and welfare problems worldwide. The pathogenic mechanism of *M. bovis* infection is still unknown, which leads to the lack of timely diagnosis and treatment. Our study found that *Mycoplasmas bovis* infection influences the host cell metabolic process and significantly represses a crucial nutritional metabolism signal pathway, mTOR. Supplement of exogenous nutrients can alleviate cell damage caused by mycoplasma. This study provides a preliminary mechanism study for *Mycoplasmas bovis* infection, which would be helpful to develop new diagnosis and treatment strategies against mycoplasma.

**Abstract:**

*Mycoplasmas bovis* (*M. bovis*) is an important pathogen that causes a variety of diseases, such as bovine respiratory diseases and causes significant losses to the national cattle industry every year, seriously affecting the development of the cattle industry worldwide. The pathogenic mechanism of *M. bovis* infection is still unknown, which leads to the lack of timely diagnosis and treatment. In this study, embryonic bovine lung (EBL) cells, infected with *M. bovis* were collected for gene profiling and detection of marker genes in the mTOR signaling pathway. The result showed that *M. bovis* infection significantly inhibits EBL growth in a dose-dependent manner. The transcription profiling data uncovered that *M. bovis* infection repressed a series of gene expressions in EBL cells, which are mainly related to metabolic process and immune response. Notably, many marker genes in the PI3K-Akt-mTOR pathway showed down-regulation after *M. bovis* infection. Further evidence showed that *M. bovis* infection inhibits expression of mTOR signaling pathway marker genes in EBL cells, which are time dependent. To further understand the *M. bovis*-induced inhibitory effect of mTOR signaling pathway, this study employed FBS as a supplement for exogenous nutrients and found that addition of a high concentration of FBS can rescue *M. bovis*-induced cell damage. In addition, a high concentration of FBS can rescue down-regulated mTOR signaling, including increasing transcriptional expression and protein phosphorylation level of mTOR pathway marker genes. This study demonstrated that *M. bovis* infection leads to inhibition of the nutrient metabolic pathway mTOR in a time-dependent manner, which would be helpful to further understand *M. bovis* infection mechanism and develop a new efficient anti-mycoplasma strategy targeting mTOR signaling.

## 1. Introduction

*Mycoplasmas bovis* (*M. bovis*), a cell-wall-less bacterium belonging to the class Mollicutes, is a devastating pathogen in dairy cows worldwide [1]. *M. bovis* infections cause a variety of disease syndromes, such as pneumonia, arthritis, mastitis and keratoconjunctivitis in feedlot cattle, dairy and veal calves and are responsible for substantial economic health and welfare problems worldwide [2]. Mycoplasmas are spherical in shape with their size varying between 0.3 and 0.8 μm in diameter and have a morphologically simple cellular structure but lack of a cell wall. *Mycoplasmas* consist of a plasma membrane, about 10 nm thick, ribosomes and a nucleoid. The plasma membrane contains a high total protein up to 50% by mass [3,4], including the variable surface proteins (Vsps) and other membrane proteins, such as P48, P68 and P26 [5]. These membrane proteins exert essential roles in *Mycoplasmas* growth, host infection and immune escape via the ability to adhere to host cells, surface antigen variation and biofilm production [6]. The control of *M. bovis* infections is still complicated due to the increasing antimicrobial resistance and a lack of effective vaccines [7]. Therefore, it remains urgent to understand the *M. bovis* pathogenesis and infection mechanisms, which will provide the basis for *M. bovis* prevention and treatment.

The ancient mechanistic target of the rapamycin (mTOR) signaling pathway coordinates eukaryotic cell growth and metabolism with environmental inputs, including nutrients and growth factors [8]. The central factor mTOR, the key downstream effector of phosphatidylinositol 3-kinase (PI3K)/protein kinase B (Akt), is a serine/threonine protein kinase whose targets include key regulators of protein synthesis [9]. mTOR can be incorporated into two protein complexes, rapamycin-sensitive mTORC1 and insensitive mTORC2. Both mTOR complexes share the catalytic mTOR subunit, the mammalian lethal with sec-13 protein 8 (mLST8), the DEP domain containing mTOR interacting protein (Deptor) and the Tti1/Tel2 complex. Other components of mTORC1 are regulatory-associated protein of mTOR (Raptor) and proline-rich AKT substrate (PRAS40), while mTORC2 contains rapamycin-insensitive companion of mTOR (Rictor) and mammalian stress-activated MAP kinase-interacting protein 1 (mSin1) [10,11]. mTORC1 is mainly involved in the regulation of the translation initiation machinery, influencing cell growth, proliferation and survival, while mTORC2 mainly participates in cytoskeleton organization, cell migration, modulation of cell cycle progression and control of cell survival [12].

Previous studies have claimed that *M. bovis* infection affects many physiological effects on host cells, including promoting apoptosis, activating endoplasmic reticulum (ER) stress, but alleviating autophagy [13,14,15,16]. Reports also raised that *M. bovis* infection induces several signaling pathways, including PI3K-Akt-mTOR pathway [17]. To further uncover pathogenic mechanism of *M. bovis* and its regulation on mTOR pathway, this study investigated gene profiling in *M. bovis*-infected embryonic bovine lung (EBL) cells and evaluated the changes in mTOR pathway under different nutritional conditions after *M. bovis* infection. Our study confirmed that *M. bovis* infection leads to inhibition of nutrient metabolic pathway mTOR in a time-dependent manner. This study attempts to help people better understand the *M. bovis* infection mechanism and develop a new efficient anti-mycoplasma strategy targeting mTOR signaling.

## 2. Materials and Methods

### 2.1. Cell Culture and Treatment

EBL cells were purchased from Fusheng Industrial (Shanghai, China) and cultured in high-sugar DMEM (Gibco, Carlsbad, CA, USA), supplemented with 10% fetal bovine serum (FBS, Gibco, Carlsbad, CA, USA). Before treatment, the cells were seeded in 6-well plates (5×10^5^ cells/well) and cultured in a condition with 5% CO_2_ at 37 °C. *M. bovis* infection assay was determined using a previously described method [18,19]. Briefly, *M. bovis* PG45 strain (preservation in our laboratory) was grown to mid-exponential phase, up to 1 × 10^9^ CFU/mL, washed three times with sterile PBS and resuspended in DMEM. The EBL cells after reaching 70% confluence were incubated with *M. bovis* at different multiplicity of infection (MOI, 5 × 10^7^ CCU/well) of 10, 20, 30, 50, 60 and 80 or different time after infection of 24 h, 48 h and 72 h, respectively. Then the cells were collected by scraping and stored for further experiments. Each treatment was conducted in triplicate and the experiments were performed three times.

### 2.2. Cell Viability Assay

EBL cells were seeded in 96-well plates at a density of 2 × 10^4^ viable cells/100 μL/well and treated with 10, 20, 30, 50, 60 and 80 MOI of *M. bovis*. The plate was then incubated in a humidified incubator at 37 °C with 5% CO_2_ for 24, 48 and 72 h. MTT (3-(4, 5-dimethyl thiazol-2yl)-2, 5-diphenyl tetrazolium bromide, Solarbio, Beijing, China) solution (10 µL) with a concentration of 5 mg/mL was added into each well to yield a final volume of 210 µL/well. Plates were incubated for 4 h at 37 °C in 5% CO_2_. After supernatants were discarded, plates added with 110 µL DMSO in each well were then placed on an orbital shaker for 10 min and the absorbance was determined at a wavelength of 490 nm using a microplate reader (Model 680, Bio-Rad, Hercules, CA, USA). Each experiment was performed in triplicate. The results are presented as percentage of the values measured for untreated control cells.

### 2.3. Transcriptional Profiling

EBL cells after *M. bovis* treatment with 50 MOI were collected for total RNA extraction and transcriptome analysis. In brief, cellular total RNAs were isolated using the Trizol reagent (Takara, Dalian, China). Qualified total RNA was further purified by an RNeasy mini kit (Qiagen, GmBH, Hilden, Germany). The mRNAs were enriched using the Oligo(dT) beads and then were fragmented and reverse transcribed into cDNA. The cDNA Library quality was checked using a Bioanalyzer2100 (Agilent, Santa Clara, CA, USA). The cDNAs were purified with QiaQuick PCR extraction kit (Qiagen, GmBH, Germany), end repaired, poly(A) added and ligated to Illumina sequencing adapters. The ligation products were size selected by agarose gel electrophoresis, PCR amplified and sequenced using Illumina HiSeq 2500 by Gene Denovo Biotechnology (Guangzhou, China). The random reads were mapped to nucleotide database Bos taurus (GCF_002263795.1_ARS-UCD1.2_genomic). The gene expression level was calculated by using the RPKM method (Reads Per kb per Million reads). FDR (False-Discovery Rate) is a method to determine the threshold of p-value. All data analyses were supported by Denovo Biotechnology (Guangzhou, China). Data were submitted to the gene expression omnibus (GEO) archive. The accession number is SUB12077437.

### 2.4. Quantitative Real-Time PCR (qPCR) Analysis

Total RNA was extracted from EBL cells using TRIzol reagent (Solarbio, Beijing, China) according to the manufacturer’s instructions. The cDNA was synthesized using a PrimeScript RT Reagent Kit (Takara, Dalian, China). qRT-PCR was performed using SYBR Green Master Mix (Vazyme, Nanjing, China) on a CFX96 Touch Real-time PCR system (Bio-Rad, Hercules, CA, USA). Target gene sequences were obtained from GenBank and quantitative primers were synthesized by Genewiz Company (Suzhou, China). The mRNA level of each tested gene was normalized to the Gapdh gene. The primer sequences used are shown as follows: Gapdh forward primer: 5′- ATCAAGTGGGGTGATGCTGG-3′, reverse primer: 5′-AGATGATGACCCTCTTGGCG-3′; mTOR forward primer: 5′-TCACCCTTGCTCTCCGAACTCTC-3′, reverse primer: 5′-TTGTGCTCGCTGTTCAGGAAGTG-3′; IGF1 forward primer: 5′- TGCGGAGACAGGGGCTTTTATTTC-3′,reverse primer: 5′- AAGCAGCACTCATCCACGATTCC-3′. These reactions were repeated three times for each sample as technical replicates. Gene mRNA quantifications were performed using the 2^–ΔΔCt^ method and the amount of transcript in each sample was normalized using Gapdh as the internal control.

### 2.5. Western Blotting Analysis

EBL cells after treatment were harvested for protein extraction using the Total protein extraction kit (KeyGen, Nanjing, China) and the protein concentration was determined by Bicinchoninic acid (BCA) kit (Beyotime, Shanghai, China). Western blotting was performed as previously described [20]. Briefly, equal total proteins were separated via 12% SDS-PAGE gel and electro-transferred to polyvinylidene difluoride (PVDF) membranes (Millipore, Bedford, MA, USA). The membrane was blocked with 10% skimmed milk at room temperature for 90 min and incubated overnight at 4 °C with the following primary antibodies: the phospho-p70 S6 Kinase Polyclonal antibody (1:1000, PA5-36864, ThermoFisher, Rockford, IL, USA) and anti-phospho-T229 S6K antibody (1:2000, ab59208, Abcam, UK). Then, following three washes with TBST twice for 5 min each time, the membranes were incubated with rabbit anti-HRP secondary antibody (1:1000, Bioss, Beijing, China) at room temperature for 1 h. Finally, bands were visualized using the Gel Imaging System (Tannon Science & Technology, Shanghai, China) and then digitized by use of ImageJ software.

### 2.6. Statistical Analysis

The numerical data were presented as mean ± standard deviation (SD) and analyzed using SPSS v19.0 statistical software (SPSS Inc., Chicago, IL, USA). The significance of the differences in each group was evaluated with a two-tailed Student’s *t*-test and *p*-values of <0.05 were considered to be significant.

## 3. Results

### 3.1. M. bovis Infection Inhibits EBL Growth

**Figure 1:**
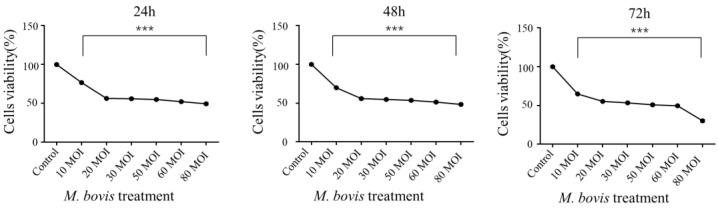
*M. bovis* infection reduced the viability of EBL cells. Cell viability after *M. bovis* infection at different multiplicity of infection (MOI) of 10, 20, 30, 50, 60 and 80 and different time of 24 h, 48 h and 72 h, respectively, were detected using MTT assay. It was determined by calculation that the half-maximal inhibitory concentration (IC50) at 24 h, 48 h and 72 h was 60, 60 and 50 MOI, respectively. The data are expressed as mean ± SEM. Experiments were repeated at least three times. *** *p* < 0.001 indicated statistical significance.

A previous study demonstrated that *M. bovis* can induce EBL cell apoptosis [14]. To further understand the biological effect of *M. bovis* on the proliferation of EBL cells and the underlying mechanisms, in the present study, EBL cell co-culture with *M. bovis* at different MOI of 10, 20, 30, 50, 60 and 80 and different times after infection of 24 h, 48 h and 72 h, respectively, were obtained for evaluation of viability rate using an MTT assay. As shown in Figure 1, it was observed that *M. bovis* infection reduced the EBL cell viability rate in a dose-dependent manner (Figure 1). *M. bovis* with 50~80 MOI reduced the EBL cells viability rate to 50% or below in 24 h, 48 h or 72 h (Figure 1). The result suggested that *M. bovis* infection significantly inhibits EBL growth. It was determined by calculation that the half-maximal inhibitory concentration (IC50) at 24 h, 48 h and 72 h were 60, 60 and 50 MOI, respectively. Further, the IC50 of 50 MOI in 72 h was determined as an indicator of *M. bovis* infection in the subsequent infection experiments.

### 3.2. M. bovis Inhibits Multiple Expression Genes for Cell Growth and Nutrient Metabolism

**Figure 2:**
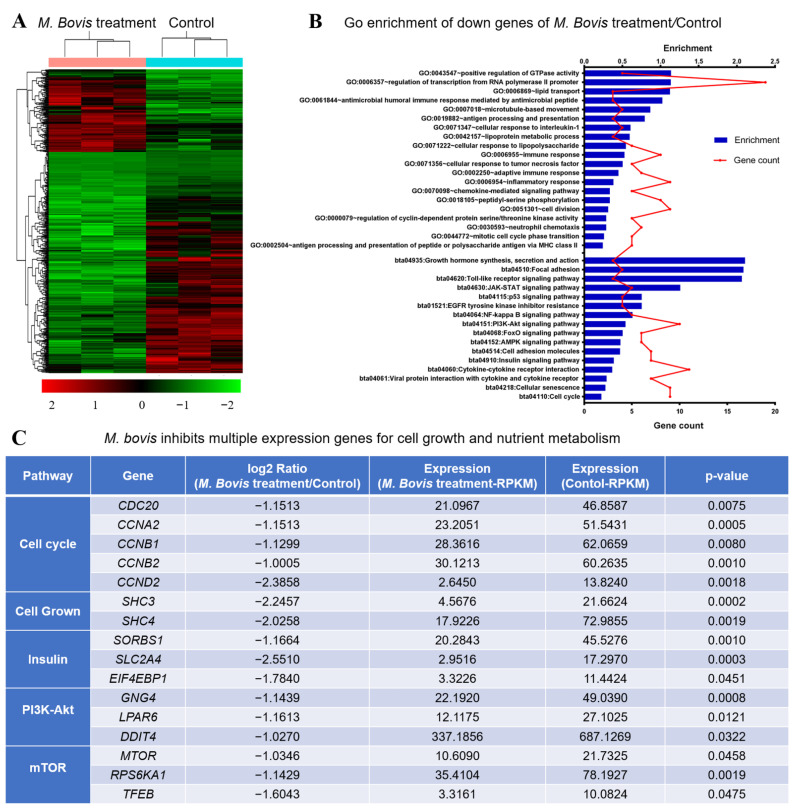
*M. bovis* inhibits multiple expression genes for cell growth and nutrient metabolism. (**A**) Heatmap showing the differences in the transcripts between the two samples, with the screening criteria of log2 Ratio of *M. bovis* infection/Control ≥1 or ≤1 and *p*-value ≤0.05. (**B**) GO annotation and KEGG pathway analysis of down-regulated genes showed that the down-regulated genes after *M. bovis* infection are mainly involved in metabolic process and immune response. Enriched factors are presented as histogram and the gene counts are shown as line chart. (**C**) Selected genes differentially regulated involved in cell cycle, cell grown, insulin, PI3K-Akt and mTOR pathways.

To gain further insights into the regulation mechanisms of *M. bovis* on EBL cells, transcription profiling of *M. bovis*-infected EBL cells was performed to search divergently expressed genes and signaling pathways. The differential genes with values of log2 Ratio (*M. bovis* infection/Control) ≥ 1 or ≤ 1 and *p*-value ≤ 0.05 were considered as significant (Figure 2). According to these criteria, it is easy to find that *M. bovis* infection results in a broad suppression of cellular gene transcription (Figure 2A). More specifically, about 390 genes were down-regulated after *M. bovis* infection, while less than 144 genes were upregulated compared to normal EBL cells. Therefore, we focused on the analysis of down-regulated genes. A detailed list of these candidate genes was provided as data (Table S1). To avoid a biased interpretation of the large number of differentially expressed genes, gene ontology (GO) annotation and Kyoto Encyclopedia of Genes and Genomes (KEGG) pathway analysis was performed to understand the enrichment of differential genes. The GO annotation revealed that the down-regulated genes by *M. bovis* infection were mainly enriched in the biological process, such as regulation of GTPase activity, lipid transport, metabolic process, antigen processing and presentation and immune response (Figure 2B). In addition, KEGG analysis showed that *M. bovis*-mediated down-regulated genes were involved in the pathways, such as growth hormone synthesis, secretion and action, Toll-like receptor signaling pathway, PI3K-Akt signaling pathway and Insulin signaling pathway (Figure 2B). Furthermore, we checked the differentially expressed genes involved in pathways, including cell cycle, cell growth, insulin, PI3K-Akt and mTOR, and found that all genes in these pathways are down-regulated by *M. bovis* infection (Figure 2C). The transcription profiling data indicated that *M. bovis* infection repressed a series of gene expressions in EBL cells, which are mainly related to metabolic process and immune response. Intriguingly, many marker genes in the PI3K-Akt-mTOR pathway showed down-regulation after *M. bovis* infection, suggesting that *M. bovis* inhibits mTOR signaling in EBL cells.

### 3.3. M. bovis Inhibit mTOR Signaling Pathway in EBL Cells

**Figure 3:**
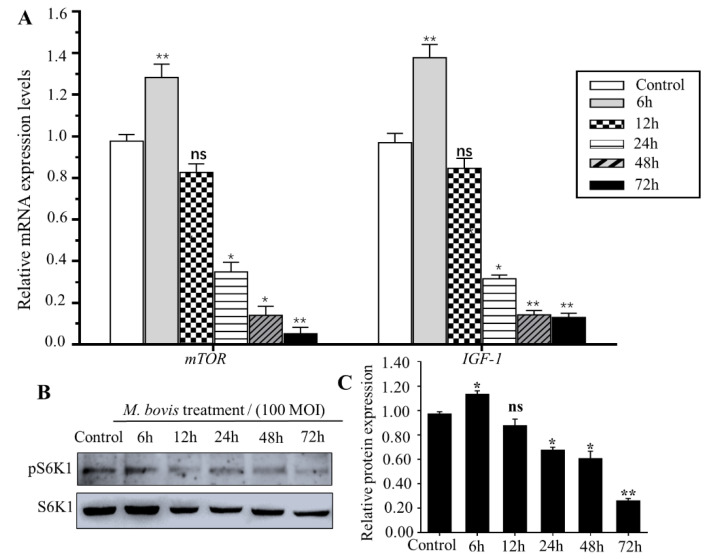
*M. bovis* inhibits mTOR signaling pathway in EBL cells. (**A**) Relative mRNA expression of mTOR marker genes, *mTOR* and *IGF-1*, was evaluated using qPCR assay after treatment with the *M. bovis* for different times in EBL cells. The mRNA levels were normalized to that of Gapdh. (**B**) Western blot analyses of phosphorylation level of S6K1 (pS6K1) expression in EBL cells after treatment with the *M. bovis* for different times. S6K1 was used as a control for the loading proteins. (**C**) The intensity of immunoblots was evaluated with ImageJ. Data are presented as the mean ± SEM of three triplicates. ns, no significant difference. * *p* < 0.05, ** *p* < 0.01 (Student’s *t*-test).

Transcription profiling showed that *M. bovis* infection inhibits mTOR signaling. Previous reports also claimed that *M. bovis* infection influenced the PI3K-Akt-mTOR pathway [17]. To further determine *M. bovis*-induced regulation on the mTOR pathway, expression of mTOR pathway marker genes, mTOR and IGF1, were evaluated using qPCR and Western blot assay after *M. bovis* infection with 50 MOI at different time points, respectively. As shown in Figure 3, after *M. bovis* infection, transcription expression changes in mTOR and IGF1 were time dependent. Transcription expression of mTOR and IGF1 increased at 6 h after infection, but gradually decreased after 12 h (Figure 3A). Expression of mTOR and IGF1 were significantly decreased at 24, 48 and 72 h after *M. bovis* infection (Figure 3A). Consistently, the phosphorylation level of S6K1, a marker of active mTOR signaling, also showed a significant decrease after 24 h with *M. bovis* infection (Figure 3B,C). These results indicated that *M. bovis* infection inhibits the mTOR signaling pathway in EBL cells.

### 3.4. High Concentration of FBS Can Save M. bovis-Induced Cell Damage

**Figure 4:**
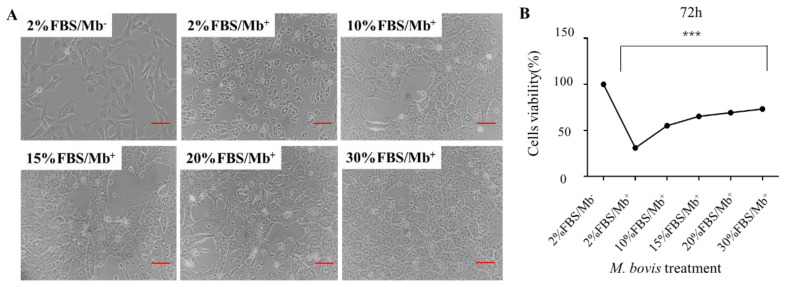
High concentration of FBS can save *M. bovis*-induced cell damage. (**A**) EBL cells after *M. bovis* infection with 50 MOI were cultured with different concentration of FBS. Cell morphology was monitored by microscopy. (**B**) Cell viability was determined by MTT assay. Scale bar = 50 μm. *** *p* < 0.001 (Student’s *t*-test).

FBS is sourced from 8–10-month-old fetal calves and contains many components conducive to cell growth, including a variety of proteins and growth factors, with the capacity to stimulate cell proliferation and the mTOR signaling pathway [21]. To further understand the *M. bovis*-induced inhibitory effect of the mTOR signaling pathway, different concentrations of FBS were added to EBL cells after *M. bovis* infection with 50 MOI. Cell morphology was monitored by microscopy and cell viability was determined by MTT assay. As shown in Figure 4, normal EBL cells were spindle shaped (Figure 4A), but after infection with *M. bovis*, the cells become round and smaller (Figure 4B). However, the addition of high-concentration FBS promoted cell proliferation and became larger (Figure 4B). EBL cells with *M. bovis* infection can be recovered to their normal shape when the FBS concentration exceeds 15% (Figure 4B). Moreover, cell viability rate can be significantly increased after *M. bovis* treatment by addition of FBS with the concentration of over 15% after 72 h (Figure 4B). The result indicated that a high concentration of FBS can rescue *M. bovis*-induced cell damage in EBL cells.

### 3.5. High Concentration of FBS Rescued mTOR Signaling

**Figure 5:**
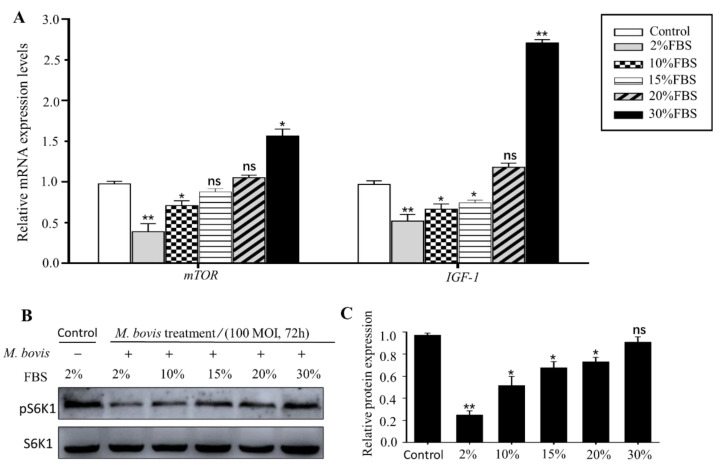
High concentration of FBS rescued mTOR signaling. (**A**) Relative mRNA expression of mTOR marker genes, *mTOR* and *IGF-1*, was evaluated using qPCR assay after *M. bovis* infection at 72 h and FBS treatment with different concentrations in EBL cells. The mRNA levels were normalized to that of Gapdh. (**B**) Western blot analyses of phosphorylation level of S6K1 expression in EBL cells after *M. bovis* infection at 72 h and FBS treatment with different concentrations. S6K1 was used as a control for the loading proteins. (**C**) The intensity of immunoblots was evaluated with ImageJ. Data are presented as the mean ± SEM of three triplicates. ns, no significant difference. * *p* < 0.05, ** *p* < 0.01 (Student’s *t*-test).

mTOR is an important signal pathway, regulating cell nutrition metabolism [8,9]. To further explore the underlying mechanism of a high concentration of FBS rescues *M. bovis*-induced cell damage, mTOR pathway marker genes were monitored after *M. bovis* infection and/or FBS treatment with different concentrations using qPCR and Western blot assay, respectively. As shown in Figure 5, *M. bovis* infection obviously decreased transcriptional expression of mTOR pathway marker genes, mTOR and IGF1 (Figure 5A). However, addition of FBS with a concentration ≥10% rescued transcriptional expression of mTOR and IGF1 and 30% FBS addition significantly increased expression of mTOR and IGF1 compared to the infection group and normal group (Figure 5A). Moreover, protein detection showed that *M. bovis* infection decreased the level of phosphorylation of S6K1, a marker of active mTOR signaling, but addition of FBS with a concentration ≥10% rescued the phosphorylation level of S6K1 (Figure 5B,C). The results demonstrated that a high concentration of FBS rescued down-regulated mTOR signaling caused by *M. bovis* infection in EBL cells.

## 4. Discussion

*M. bovis* is an important pathogen that causes a variety of disease syndromes, such as pneumonia, arthritis, mastitis and keratoconjunctivitis, in feedlot cattle, dairy and veal calves and causes significant losses to the dairy cow industry, seriously affecting the development of the cattle industry worldwide [1,2]. Due to the increasing antimicrobial resistance and a lack of effective vaccines, the control of *M. bovis* infections is still complicated [7]. It remains urgent to understand the *M. bovis* pathogenesis and infection mechanisms, which will provide the basis for *M. bovis* prevention and treatment. Previous studies claimed that *M. bovis* infection affects many physiological effects on host cells, including promoting apoptosis, activating endoplasmic reticulum (ER) stress and alleviating autophagy [13,14,15,16]. However, the knowledge about the *M. bovi*s-regulated signaling pathway is still limited.

Our study confirmed that *M. bovis* infection significantly inhibits EBL growth in a dose-dependent manner. The transcription profiling data uncovered that *M. bovis* infection repressed a series of gene expressions in EBL cells, which are mainly related to metabolic process and immune response. Intriguingly, many marker genes in the PI3K-Akt-mTOR pathway showed down-regulation after *M. bovis* infection, suggesting that *M. bovis* inhibits mTOR signaling in EBL cells. Further evidence showed that *M. bovis* infection inhibits expression of mTOR signaling pathway marker genes in EBL cells, which are time dependent. Consistently, a previous study reported that *M. bovis* inhibits autophagy via a PI3K-Akt-mTOR-dependent pathway [17], which also showed that mTOR signal was stimulated at 6 h after *M. bovis* infection, but gradually decreased after 12 h.

The mTOR signaling pathway coordinates eukaryotic cell growth and metabolism with environmental inputs, including nutrients and growth factors, and exerts an essential role in regulating cell nutrition metabolism [8,22]. This study employed FBS as a supplement of exogenous nutrients and found that the addition of a high concentration of FBS can rescue *M. bovis*-induced cell damage. In addition, a high concentration of FBS can rescue down-regulated mTOR signaling, including increasing transcriptional expression and protein phosphorylation level of mTOR pathway marker genes. This study demonstrated that *M. bovis* infection leads to inhibition of the nutrient metabolic pathway mTOR in a time-dependent manner, which gives us the enlightenment that activation of the host mTOR signal pathway can antagonize *M. bovis* infection. This study would be helpful for people to develop new efficient anti-mycoplasma strategies targeting mTOR signaling via activating mTOR pathway effectors.

## 5. Conclusions

Our study found that *M. bovis* infection significantly inhibits EBL growth in a dose-dependent manner. The transcription profiling data uncovered that *M. bovis* infection repressed a series of gene expressions in EBL cells, which are mainly related to metabolic process and immune response. Notably, many marker genes in the PI3K-Akt-mTOR pathway showed down-regulation after *M. bovis* infection. Further evidence showed that *M. bovis* infection inhibits expression of mTOR signaling pathway marker genes in EBL cells, which are time dependent. However, addition of a high concentration of FBS can rescue *M. bovis*-induced cell damage and down-regulated mTOR signaling. This study demonstrated that *M. bovis* infection leads to inhibition of nutrient metabolic pathway mTOR in a time-dependent manner, which would help to further understand the *M. bovis* infection mechanism and develop a new efficient anti-mycoplasma strategy.

## Data Availability

The raw data of gene profiling were submitted to the GEO archive with link: https://www.ncbi.nlm.nih.gov/sra/PRJNA882585 (accessed on 25 September 2022).

## Supplementary Materials

The following supporting information can be downloaded at: https://www.mdpi.com/article/10.3390/vetsci9110630/s1, Table S1: Differentially expressed genes.
